# Massachusetts Dental Society

**Published:** 1899-12

**Authors:** 


### MASSACHUSETTS DENTAL SOCIETY.

(Continued from page 759.)

### INFECTIOUS DENTISTRY.

BY ANDREW J. FLANAGAN, D.D.S., SPRINGFIELD, MASS.

BY the title of this paper my audience may think that here is another one of those papers dealing with the great "bacterial question," a long list of infallible remedies, and last, but not by any means least, a long list of unhappy results, and even deaths owing to the inability of the individual to master the pathogenic forms.

We who have read the dental and medical journals for the last ten years have noticed that all that which can be gathered under the one word "antisepsis" has been of vast amount, and presented in such a form that we may have had a veritable bacteria nightmare at times. Now, while dentists have advanced in this line, the public also has kept apace, for the public press has fairly teemed with matter relative to this subject. Journalism of the present day is of such a broad character that we read much that a few years ago was confined to technical journals. Such being the case, it behooves us to so practise our calling that there will be no question as to its cleanliness in the minds of the laity. The Commonwealth of Massachusetts to-day offers a field for the intelligent practice of dentistry surpassed by none; we see every year a goodly increase in our number here, and the vast majority seem to gain a fair practice, and it is fair to presume that this increase will still continue, for the law of demand and supply will ever be the means of regulation. In order that this writing may be of a logical nature, I will define infections as that which *may* infect, contaminate, vitiate, or corrupt. Understand the word *may* is used; and those who are of a sensitive nature in discussions of this kind may take refuge accordingly. There is also a little quotation I would have you carry in your minds,—"If we could but see ourselves as others see us."

For the last three years it has been my pleasure to be associated with many physicians while acting as a member of the House of Mercy Hospital staff in the city of Springfield. In that time we have had several cases where infection *may* have been the cause of the trouble. In tracing out the history of one of these my eyes were so opened that it was decided to look thoroughly into the question of "antisepsis" as practised by the average dentist. The saliva-ejector is a most useful adjunct, but it needs great care to keep it in a thoroughly clean condition. A short time ago a student in an office in our city pointed to my ejector, and said he would not have that used in his mouth. Being questioned for a reason, he said the doctor always tended to the cleaning of this, and he had seen him use it time after time on different patients without boiling. It has been found that burs, clamps, and broaches do not get the care that excavators, etc., do. The use of modelling compound the second time presents food for thought. How can it be sterilized? Brushes, cups, and disks used in cleansing the teeth are often used again without sterilizing, even after dipping into blood and pus. Are we always careful in the use of our stones for grinding in the mouth? Many teeth are extracted and patients sent from the office without instruction as to subsequent care. It is common for these cases to afterwards fall into the hands of reputable physicians for treatment. I have been astonished to hear the criticisms given by intelligent people on certain procedures in our every-day work, and this thought has presented itself,—how long can the public be fooled without disastrous results to the good name of dentistry?

How common it is to hear dental practitioners deride the knowledge the average physician seems to possess of lesions in the oral cavity. We had best remove the scales from our eyes when this criticism extends to that which we will call antisepsis. The writer has always found the hand of friendship extended from the ranks of medicine, and the true worth of dentistry acknowledged. Not long since a bright M.D. asked me how it was that he had seen so much hypertrophy of the gums in his practice; the patients had been to the dentists for treatment, and in every case said treatment consisted in the removal of salivary calculus and polishing the teeth. How common it is to see beautiful fillings and crown-work, and yet the soft tissues of that mouth in a diseased condition! How can our patients have good digestion and a fair condition of the breath where such conditions exist? How common to hear at our meetings the wish for papers and clinics of a more practical nature! A clinic given on the common diseased conditions of the mouth would be of inestimable value, and might lessen in the future the number seeking relief by the service of the physician, for it is in the domain of pathology and surgery that the future of dentistry rests.

We have had our age of the tooth-carpenter, and we will now have that of the tooth-doctor. Then we will not hear practitioners expatiate on how many teeth they can extract in a second; how many books of gold they can condense in a half-hour; and how they can beat the handiwork of the Creator by having the strain and stress of fourteen teeth done by four. Gentlemen, this is the period of prophylaxis, and I trust that we may live to see dentistry broadened to its true professional proportions. The fountain head is not higher than its source, then let us each strive for that breadth of knowledge which hinders not but hastens development.

DISCUSSION OF DR. FLANAGAN'S PAPER.

*Dr. N. Morgan* (Springfield, Mass.).—I do not care to say much now, but I would like to inquire of Dr. Flanagan what he would suggest in the way of antiseptic dentistry.

*Dr. A. J. Flanagan.*—Well, I will tell you why I presented that paper. It is not a technical paper; it is simply a statement of facts which have come under my personal observation in the last three years. My idea of dentistry is this: you cannot make a profession of it unless you have professional members practising it. Why is it that the average dentist does not follow correct asepsis in his practice? He is being observed by intelligent people, people, in some instances, without a very high idea of dentistry, I think there are more dentists to the number of people in Massachusetts than in any other State. You will find that there is not the feeling between the medical profession and dentists that there should be, and if you will get a friendly physician in the right condition, he will tell you why he thinks we are weak. Now take the people who do extracting; a patient will go to them and they will extract a tooth, and that tooth is in an unhealthy condition, or the gums are from which the tooth is taken. They will extract the tooth, get their half-dollar, and the transaction is ended. The doctor does not give the patient a mouth-wash or instruct him to come back and have the cavity treated, but he is allowed to leave the office, inflammation sets in, and the patient, fearing serious results, calls in a regular M.D.

We will take the condition of diseased gums as we find them presented to the medical practitioner. He examines the mouth, and finds that the patient has been in the hands of a reputable dentist, and yet the gums have been left in a diseased condition. Yet we claim to be doctors of dental surgery. If there is hypertrophy of the gums, what does the ordinary dental practitioner do? He may place a little iodine on it, but how very seldom is a lancet or a pair of shears taken to remove it. Now, there is no reason why we should not do it rather than leave it for a physician to do afterwards. It is a common thing for a physician to have a practice among dentists of administering ether. He goes into their laboratories, he sees the dentist come in after treating a patient, and at times he observes the dentist using the same instruments over again which have been used in treating a patient, and they have even been immersed in blood and pus, and the sterilizing consists in immersion in cold water and wiping with a soiled towel or napkin. I find that there is a good deal of truth in the statement that we do not always attend to our instruments as we should. The question of modelling compound has raised quite a little trouble. One of the physicians up our way recently asked me if I ever used modelling compound a second time. I told him I did. "How do you sterilize it?" You cannot boil it. The only way you can sterilize it is to take a solution of bichloride of mercury, or something of equal power. That called to my mind the fact that I was doing something that was not right. Since then I have sterilized my modelling compound when using it a second time. I am not going to tell you how to sterilize instruments, for you all know, even if you do not practise it. I boil my instruments where it is possible, and when not, I use bichloride of mercury in solution. My paper was not to tell you how to sterilize, but to tell you that we do not take proper care in keeping our instruments thoroughly clean.

### TURPENTINE.

BY R. H. CLARK, D.D.S., NORTHAMPTON, MASS.

I have not selected for your consideration a new subject, but one the name of which is familiar to all, and its specific properties are of great value in many dental operations.

I can make no claim for originality for the applications of turpentine, except to apply to conditions similar to those treated by our friends of the medical profession. Some of these have, so far as any previous knowledge of drugs is concerned, been unknown to me, and thus may be original.

If there is a question in all the realm of our profession so perplexing as inflammation, and how best treated to insure comfort to the patient and gratifying success to the operator, it has not appealed thus to me. My preliminary education in dental branches was inadequate to answer the question when confronted by it, and only by a conscientious determination to acquire knowledge have I been partially able to fulfil my duties.

I do not claim for turpentine that it is the most valuable agent, but make the claim that it stands without a peer in alleviating certain conditions to be described hereafter.

Turpentine is of strong, diffusive odor, hot, pungent taste, non-corrosive, antiseptic, germicidal, preservative, an antiferment and disinfectant, and is practically non-poisonous, because adults have been known to have taken from four to six ounces without loss of life. Howbeit, overdoses taken internally produce nausea and thirst, a febrile state is induced, muscular strength reduced, coordination impaired, strangury develops, and finally profound insensibility and abolition of all reflex movements. It is obtained from species of pine, and by distillation the oil is produced. It readily absorbs oxygen, which it retains with great tenacity, hence enhancing its value as a remedy. Its action, when applied to skin or mucous surfaces is counterirritant, rubefacient, and anodyne. Its physiological action produces heat and redness followed by vesicular eruption and sometimes ulcerations. The heart's action is increased, arterial tension arises, a general sense of warmth and exhilaration of parts are experienced. It stimulates the vasomotor nervous system, exhausts the irritability of sympathetic ganglia, and stimulates respiration.

As an antiseptic, medical experts are satisfied that it is a product which produces a beneficial physiological action, and many thorough and painstaking investigators, in hospital and private practice, have established its claim to a very prominent place in the field of antiseptics, because in surgical dressings a thorough antisepsis of the wound's discharge is effected.

It is also given internally to combat many distressing and serious diseases, as cholera, disease of lungs, etc. In germicidal properties it ranks second to bichloride of mercury, and is preferable because of its harmless nature. It prevents fermentation, and in the arts is invaluable on account of its great preserving properties. In dental therapy we find use in the following pathological condition: It counteracts the after-pain of tooth extraction. It is valuable as a root-canal dressing, the penetrating properties of the oil being carried throughout the canals of roots, even to the apices, thoroughly antisepticizing and rendering, finally, by its irritating nature the incipient abscess (if one) as nil. In periostitic troubles it is no doubt without a parallel. By its counterirritating properties that acute inflamed condition becomes quieted, and smiles of appreciation illumine the faces of our patients.

In cases like the above its action is peculiar according to the vitality of the patient. Sometimes it acts as a resolvent, again it hastens (or seems to) ulceration.

In leucoplakia, in scurvy, or those turbulent conditions evolving hypertrophy of the gums, it acts quickly, quietly, and very effectively.

In mercurial stomatitis it has no superior. Let me emphasize its use to this pathological condition. In at least half a dozen cases in my practice it has never failed as yet to perform that for which it is intended. In operations for necrosis it acts to relieve that condition of intense pain.

It is an antidote for phosphorus, preventing the formation of phosphoric acid, and converts the substance into an insoluble spermaceti-like substance. Thus it is indicated in the phosphoric necrotic condition in mouths of those working in match-factories.

In all cases it tends to arrest the hemorrhagic exudations, fermentation, and putrefaction processes, with but little pain attending its application.

As a counterirritant we use it thus: To one part turpentine add one part aconite and two parts iodine. Apply directly to afflicted parts by means of a cotton swab saturated with it.

In hypertrophic conditions, remove all irritated surfaces; apply oil of turpentine in such strength as is practicable.

If I have revived in your memory the application of one of the most useful of remedies, the trip to this convention has not been in vain, for I can assure you, if you will use it you will agree with me that its value has not been overstated.

### DISCUSSION OF DR. CLARK'S PAPER.

*President H. S. Draper.*—Gentlemen, Dr. Clark's paper is before you for discussion. The remedy in connection with dentistry is new to me, but if there are any who have tried it, we should be pleased to hear from them.

*Dr. Geo. A. Maxfield* (Holyoke, Mass.).—I have not had any experience with the use of turpentine personally, but last Decoration Day I had the pleasure of riding with an old veteran who had lost a leg in the Civil War. He told me his experiences as to how he lost the leg, how the surgeons used the same sponge for from fifty to a hundred men, and how his leg, where the amputation had been performed, first mortified, and then became a mass of maggots. The surgeons told him that he was threatened with gangrene, and that it was doubtful if he would ever recover. Finally, he bribed one of the nurses to bring him some turpentine, and succeeded in procuring about a pint. He took the stump of his leg and poured the turpentine in, and in less than an hour he poured out over a pint of dead maggots. When the surgeon came around again he said, "What has been going on here?" He said, "I have been washing it out with turpentine." He used it for about three days, when the surgeon said he could send him home in a few days, and in three weeks' time it was healed.

I cannot explain why this well-known remedy should have passed out of use. I am glad Dr. Clark has brought this before us, and I hope more study and experiment will be given to it.

*Dr. J. King Knight* (Hyde Park, Mass.).—Dr. Clark has used the expression "oil of turpentine." Does he mean the oil or the spirits?

*Dr. R. H. Clark.*—I do not know of the different kinds of turpentine. It is not dissolved very readily in water, but it makes a mixture that you can readily use. When using it I mix it with warm water.

A young lady, about sixteen, with a fistula that had been treated by a doctor of medicine, came to me for relief. I removed the sixth-year molar, and treated the socket just once. I then went to Michigan, returning Monday, and saw this case, and it was well.

*President Draper.*—I would like to ask if this has been used in cases of empyema of the antrum.

*Dr. R. H. Clark.*—I never have used it for that.

*Dr. B. H. Stout* (Taunton, Mass.).—Dr. Clark in his original paper suggests a combination of turpentine, aconite, and iodine. Was this mixture used in this instance in his treatment of the sixth-year molar? If so, it seems to me Dr. Clark lays too much stress on the value of turpentine and not enough on the other two ingredients. If he had washed out the mouth with water, would not the result have been the same?

I think it is one of those things which must be used with caution, and I should want to know the authority for the statement that turpentine stands next to bichloride of mercury as a disinfectant. I do not think the medical profession use turpentine as a disinfectant, for I think there are other and milder remedies which are more powerful and better germicides than it.

*Dr. G. A. Maxfield.*—I know a few years ago a remedy for peritonitis was turpentine stupes. They would wring out a cloth from hot water, sprinkle it with turpentine, and put it on the inflamed parts.

*Dr. B. H. Stout.*—A turpentine stupe is an old remedy, and probably would not now be used in peritonitis.

*Dr. Lewis S. Breed* (Rosindale, Mass.).—Two years ago I was in the Massachusetts General Hospital for two months, with a case of appendicitis, and for four days after the operation I was in fearful pain. They gave me turpentine enemas, which gave me such relief that I called for them much oftener than they would give them to me. I have ever since blessed turpentine.

*Dr. W. I. Brigham* (South Framingham, Mass.).—There was a man I knew who had a tapeworm. They gave him oil of turpentine, and it killed the tapeworm.

*Dr. S. G. Stevens* (Boston, Mass.).—Was it the oil or the spirit?

*Dr. R. H. Clark.*—I have always used the oil of turpentine.

*Dr. S. G. Stevens.*—Did you ever give a patient turpentine to take internally?

*Dr. R. H. Clark.*—The preparation I have used in dentistry as a wash is a mixed condition (because of its insoluble properties). I have used it with iodine and aconite. I admit that we use iodine and aconite in fixed and definite proportions of each, but if you use turpentine it has a better effect. It was given to me in Michigan as a suggestion. The wash I have used is just as strong as the patient can comfortably bear. I assure you, gentlemen, that it is a benefit to use it.

*Dr. S. G. Stevens.*—I have not received an answer to my question. I do not know whether we are using oil of turpentine or spirit of turpentine.

*Dr. W. I. Brigham.*—If you buy oil of turpentine, you get spirit of turpentine. If you buy spirit of turpentine, you get oil of turpentine. They are both the same.

*Dr. Geo. A. Maxfield.*—You know it is very unsafe for a pregnant woman to go into a room that has been recently painted. I do not know that it is the turpentine in the paint that causes the trouble, or the lead or other matters used, as I have not experimented very much with turpentine. We ought to be well informed on that question.

Dr. George T. Baker then read his paper on "Dental Surgeons in the Army and Navy."

(For Dr. Baker's paper, see page 773.)

DISCUSSION.

*Dr. J. King Knight* (Hyde Park, Mass.).—I believe that every one here believes emphatically that the army and navy should contain a dental corps without any question. I cannot think that any intelligent dentist believes otherwise, and with that in view, I move that resolutions be passed here and forwarded to our Congressional representative. We should have a representation in the army and navy. They are one hundred years behind the times.

The motion was seconded and the question put by the President and carried.

*Dr. J. King Knight.*—My idea was that a copy of that resolution should be sent to our Massachusetts Congressman, and then all those professional wire-pullers are to use their personal influence.

*Dr. A. J. Flanagan.*—It has been my experience that when you send a letter through the mail to a Congressman, it sometimes reaches the waste-paper basket. He will ask the question, "Who are the members of this Massachusetts Dental Society? Have they any votes? Now, in my opinion it would be a better idea to appoint three men, good practical wire-pullers, and I would move you that a committee of three—but with the understanding that the speaker is not to be a member of said committee—be appointed, to take this matter and do as they see fit. I offer this as an amendment to Dr. Knight's motion.

*Dr. Carl R. Lindstrom.*—Do I understand that there has been a time set when this committee shall report?

*Dr. A. J. Flanagan.*—My idea was that it start immediately on its work and make its report at the next meeting.

*Dr. D. Hurlburt Allis.*—Have we not a Legislative Committee for this purpose?

*President Draper.*—The Legislative Committee is not for this purpose, but if there be no objection they might take this on their hands. Will Dr. Flanagan state how he wishes this committee appointed?

*Dr. A. J. Flanagan.*—I do not care how the committee is appointed, but I do not think a committee of five is as good as a committee of three, and I think the chair should do the appointing.

(To be continued.)

